# A Novel Method of Treatment of Macrodactyly with Digital Nerve Resection and Nerve Allograft

**DOI:** 10.1097/GOX.0000000000002483

**Published:** 2019-10-29

**Authors:** Edward M. Kobraei, Marie N. Dusch, Erin M. Meisel, Milan Stevanovic

**Affiliations:** From the Department of Orthopedic Surgery, Children’s Hospital Los Angeles, University of Southern California Keck School of Medicine, Los Angeles, Calif.

## Abstract

Macrodactyly is a rare and debilitating pediatric hand anomaly that has historically been treated with amputation. Recent advances in our understanding of macrodactyly have increasingly implicated the digital nerve as the underlying cause of the condition. In addition, much progress has been made toward digital salvage in treating macrodactyly. Modern techniques often advocate for digital nerve resection to prevent recurrence and treat the underlying etiology. However, little if any emphasis has been given to sensory reconstruction for purposes of protective sensation and acceptable 2-point discrimination at the volar digital pulp. We report 2 cases of macrodactyly treated with digital nerve resection and nerve allograft reconstruction of large sensory nerve gaps which show early promising sensory outcomes. There remain little if any data on the use of nerve allograft in pediatric populations, and its application in the treatment of macrodactyly. This technique permits digit salvage with the potential for improved sensory outcomes without the donor morbidity of autologous nerve harvest.

## Abstract 2

Supplemental Digital Content is available in the text.

## INTRODUCTION

Macrodactyly is a heterogeneous and often sporadic condition involving less than 1% of all congenital upper extremity conditions.^1^ Despite its rarity, the condition remains a daunting surgical challenge with uncertain etiopathogenesis. The goals of treatment include functional digits with mobile interphalangeal joints, excellent 2-point discrimination, and acceptable aesthetic appearance. Numerous surgical approaches have been described including soft tissue and skeletal reduction, epiphysiodesis for adult-sized digits, partial amputation, ray amputation, and digit or toe free tissue transfer.^2–5^ Results in these cases have been frequently suboptimal and unpredictable.

Recent advances have been made in our understanding of macrodactyly. Among the most seminal of these has been discovering the critical role of PIK3CA activating mutations in conditions of overgrowth like macrodactyly.^6,7^ In the most common form of macrodactyly, the characteristic clinical finding is digital overgrowth defined specifically to the dedicated nerve territory of a digital nerve. This has often been differentiated in various classification schemes as “nerve territory-oriented macrodactyly.”^5,8,9^ In these cases, the clinical and pathologic findings of the digital nerve include fatty infiltration, enlargement, and tortuosity of the nerve.^5^

With increasing implication of the digital nerve in the etiology of macrodactyly, there is renewed interest in developing surgical treatments guided by an etiopathological approach. Procedures for nerve territory-oriented macrodactyly have emphasized digital nerve preservation or have advocated nerve resection with little if any consideration of sensory reconstruction.^2–5,8^ We have incorporated a novel approach in the treatment of macrodactyly involving targeted digital nerve resection, soft tissue debulking, and nerve allograft for sensory nerve reconstruction. Our early experience suggests that this approach can yield aesthetic and functional digits with promising early sensory outcomes.

## CASE EXAMPLES

**Fig. 1.**
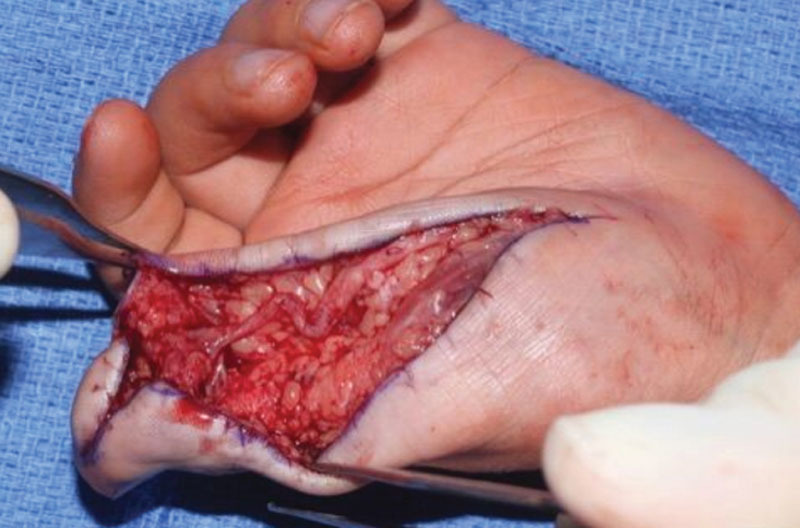
The radial digital nerve of the thumb was found to be tortuous and hypertrophic.

Case 1: A 4-year-old otherwise healthy male patient with progressive left thumb and thenar macrodactyly was referred to the senior author. The tissue overgrowth was predominately limited to the thenar compartment and radial digital nerve distribution of the thumb (**see figure, Supplemental Digital Content 1**, which displays preoperative view of patient with macrodactyly in the radial digital nerve distribution of the thumb, **http://links.lww.com/PRSGO/B237**). The patient underwent segmental resection of the radial digital nerve with radical debulking of the radial side of the thumb and thenar area. Intraoperatively, the radial digital nerve was found to be thickened and tortuous, with extensive fibrofatty infiltration (Fig. 1). The nerve gap was reconstructed with a 1.5-mm (diameter) × 40-mm (length) interposition nerve allograft for tension-free sensory reconstruction. At 8 months of follow-up postoperatively, the patient had 4-mm 2-point discrimination of the radial thumb pulp tested by 2 certified hand therapists (**see figure, Supplemental Digital Content 2**, which displays a 4-cm interposition nerve allograft that was used for tension-free sensory reconstruction, **http://links.lww.com/PRSGO/B238**); the patient had recovered 4-mm 2-point discrimination of the radial volar pulp of the thumb (**see figure, Supplemental Digital Content 3**, which displays 8-month postoperative palmar view of the hand, **http://links.lww.com/PRSGO/B239**); and the patient had recovered 4-mm 2-point discrimination of the radial volar pulp of the thumb (**see figure, Supplemental Digital Content 4**, which displays 8-month postoperative dorsal view of the hand, **http://links.lww.com/PRSGO/B240**).

**Fig. 2.**
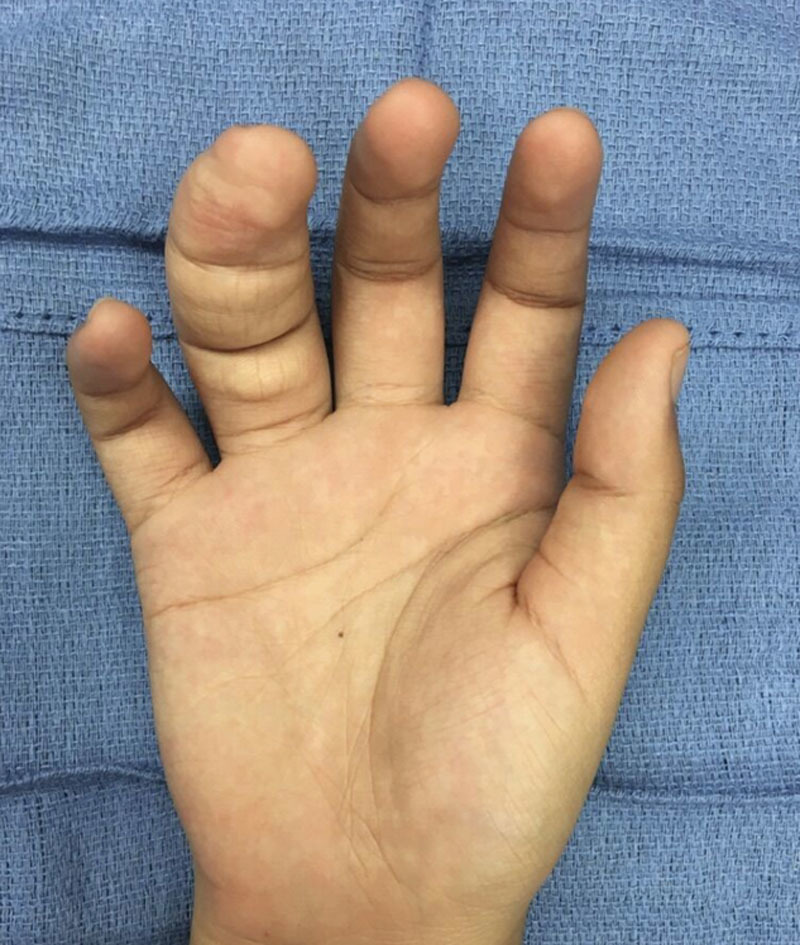
Preoperative palmar view of the hand in a patient with macrodactyly in the ulnar digital nerve distribution of the ring finger.

**Fig. 3.**
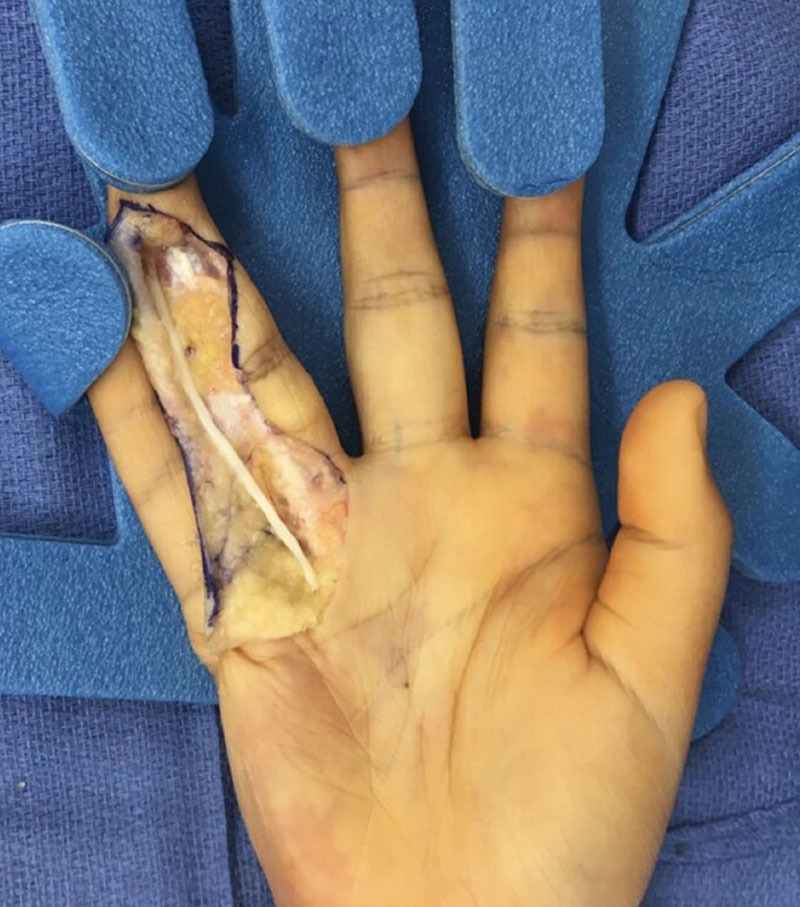
After ulnar digital nerve resection, the resulting 7-cm nerve gap is reconstructed with nerve allograft.

**Fig. 4.**
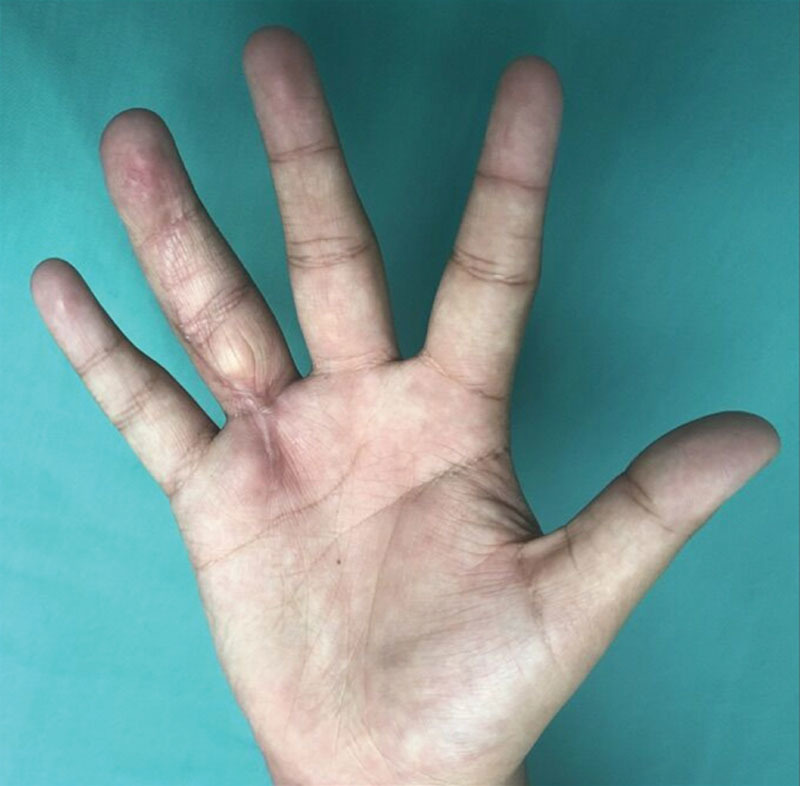
Six-month postoperative palmar view of the hand. The patient has normal function and a migrating Tinel’s sign along the ulnar digital nerve distribution, with no return of sensation of ulnar volar digital pulp at this time.

Case 2: A 12-year-old otherwise healthy female patient with static, predominately ulnar-sided right ring finger macrodactyly was referred to the senior author (Fig. 2). The patient underwent ulnar digital nerve resection and debulking followed by nerve reconstruction using a 1.5-mm (diameter) × 70-mm interposition nerve allograft (Fig. 3). At 6 months postoperatively, the patient was noted to have excellent function and a migrating Tinel’s sign in the ulnar digital nerve distribution without appreciable sensory return at the level of the volar digital pulp (Fig. 4).

## DISCUSSION

Historically, amputation was often the treatment of choice for macrodactyly. Treatment strategies have since been refined with increased understanding of the disease pathophysiology. Due to the heterogeneity of the disease, no single treatment strategy has proven universally effective or applies to all cases.

Tsuge first popularized the theory that the hypertrophic nerve is the driving force for digital overgrowth in macrodactyly.^3^ Gluck and Ezaki further emphasized the need for complete nerve excision due to nerve-driven overgrowth of the digit, which is thought to be mediated by diffusible growth factors.^2^ More recent treatment strategies advocate for complete excision of the involved hypertrophic nerve along with the surrounding soft tissue. On the other hand, others advocate for a nerve-sparing approach, which provides a sensate digit but unfortunately fails to eliminate the cause of the overgrowth. There are few if any reports on sensory reconstruction following digital nerve resection in the treatment of macrodactyly.

Increasing emphasis on both nerve resection and digit salvage has resulted in the need to develop methods of sensory reconstruction with the goal of protective sensation and acceptable 2-point discrimination at the level of the volar digital pulp. We demonstrate 2 cases of our novel method of targeted, radical resection of the involved digital nerve with the use of nerve allograft for sensory reconstruction. Our early experience has yielded aesthetic digits with improved sensation in the early postoperative period.

In this technique, we perform a radical resection of the digital nerve, removing all digital nerve that is grossly diseased until a healthy proximal nerve stump is identified. This requires dissecting the digital nerve proximally to a level outside the gross overgrowth and, therefore, may result in sensory nerve gaps that are quite large. A distal nerve stump is left in place for purposes of volar pulp sensory reconstruction using processed nerve allograft.

Our preference for managing this nerve gap is for nerve allograft over autologous nerve. Nerve allograft is often immediately available, results in shorter operative times, and avoids the donor morbidity of autologous nerve harvest including the possibility of an additional sensory-deficit or painful traumatic neuroma. To our knowledge, there are no publications available that incorporate nerve allograft in the treatment of macrodactyly. Much of the available data to date on sensory outcomes with allograft comes from the adult trauma population.^10^ In comparison with the use of nerve conduits, nerve allografts have improved functional and sensory outcomes.^11^ In a multicenter registry study, sensory outcomes for nerve allografts were equivalent to historical controls for nerve autograft and exceeded those of conduits when used for short-gap digital nerve reconstruction.^12^ With regard to injuries greater than 25 mm, a study by Rinker et al found favorable sensory outcomes using nerve allograft in gap lengths up to 50 mm when compared with historical reports for nerve autograft, but without donor-site morbidity.^13^ Pediatric macrodactyly patients have the potential for optimal outcomes using nerve allograft given their age and use in a nontrauma setting.

This technique has its limitations. It is best suited to cases of digital overgrowth confined to the distribution of a single digital nerve, as is often the case. We would exercise caution applying this to cases where both digital nerves are involved clinically, as the digital arteries may be quite hypoplastic. We acknowledge the limitation and accuracy of testing for 2-point discrimination in a young child. Longer-term follow-up is needed to fully ascertain final sensory function such as 2-point discrimination, protective sensation, and the possibility of disease recurrence given the historically high disease recurrence in children with macrodactyly with existing surgical approaches. There is a potential, although it is unknown at this time, that grafting of the diseased nerve could cause earlier disease recurrence. In cases where diseased nerve exists distally, we resect the digital nerve to a level just proximal to the distal interphalangeal joint, to permit an adequate stump for neurorrhaphy and to provide sensory reconstruction to as much digital pulp as possible. Leaving some diseased nerve distally may influence sensory return or recurrence of macrodactyly, and this may prove to be a limitation of this technique.

In addition, nerve allograft may be prohibitively expensive or unavailable at certain centers and judicious use of this technique should be exercised, perhaps prioritizing critical sensory territories such as that of the thumb, border digits, and radial side of the index finger.

## Supplementary Material
