# Supplementary figures and images for: A Novel Method of Treatment of Macrodactyly with Digital Nerve Resection and Nerve Allograft
Source: Plast Reconstr Surg Glob Open. 2019 Oct 29;7(10):e2483. doi: 10.1097/GOX.0000000000002483 (PMC6846287; doi:10.1097/GOX.0000000000002483)

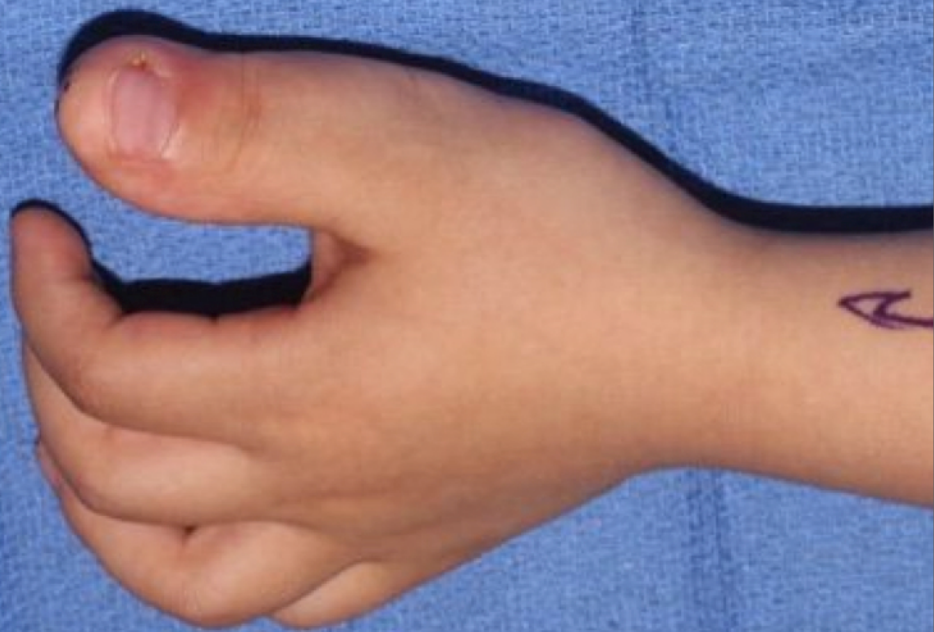

Supplement: Supplementary file 1 [file gox-7-e2483-s001.pdf]

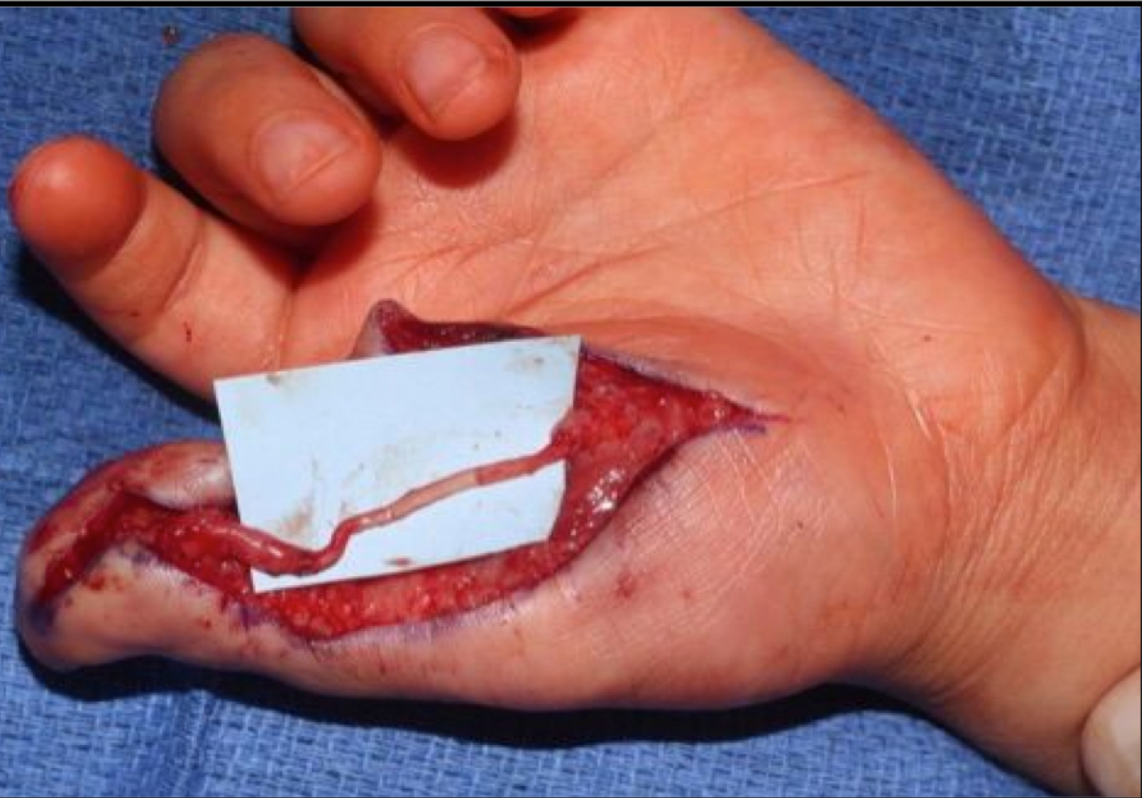

Supplement: Supplementary file 2 [file gox-7-e2483-s002.pdf]

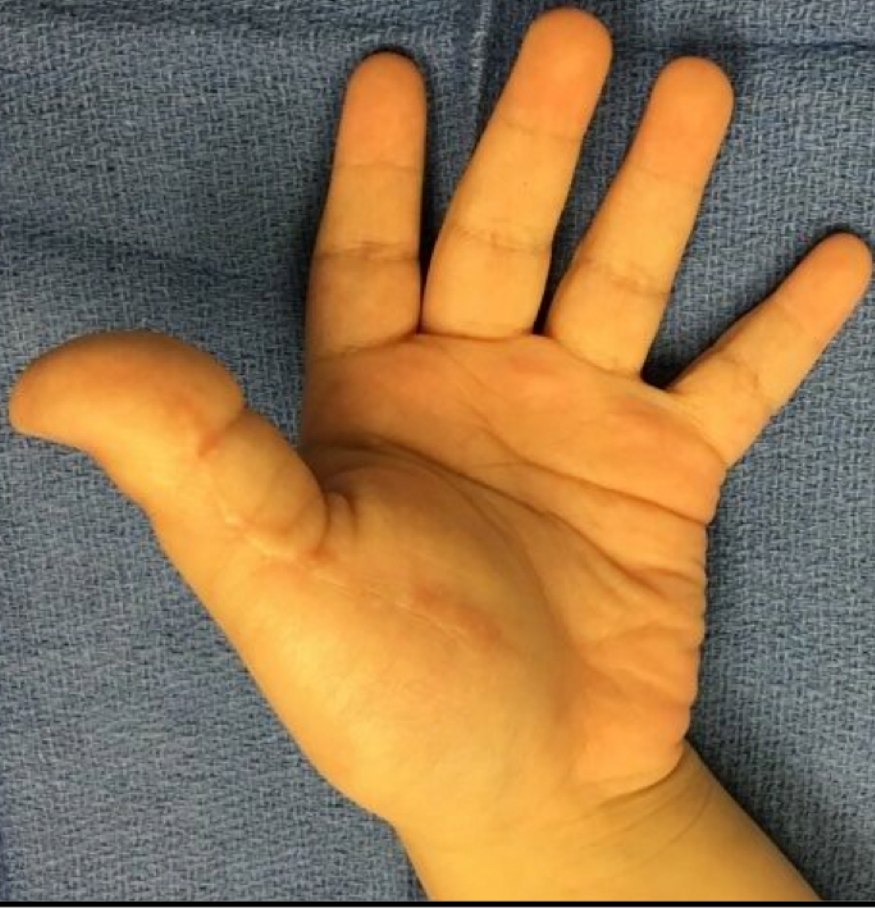

Supplement: Supplementary file 3 [file gox-7-e2483-s003.pdf]

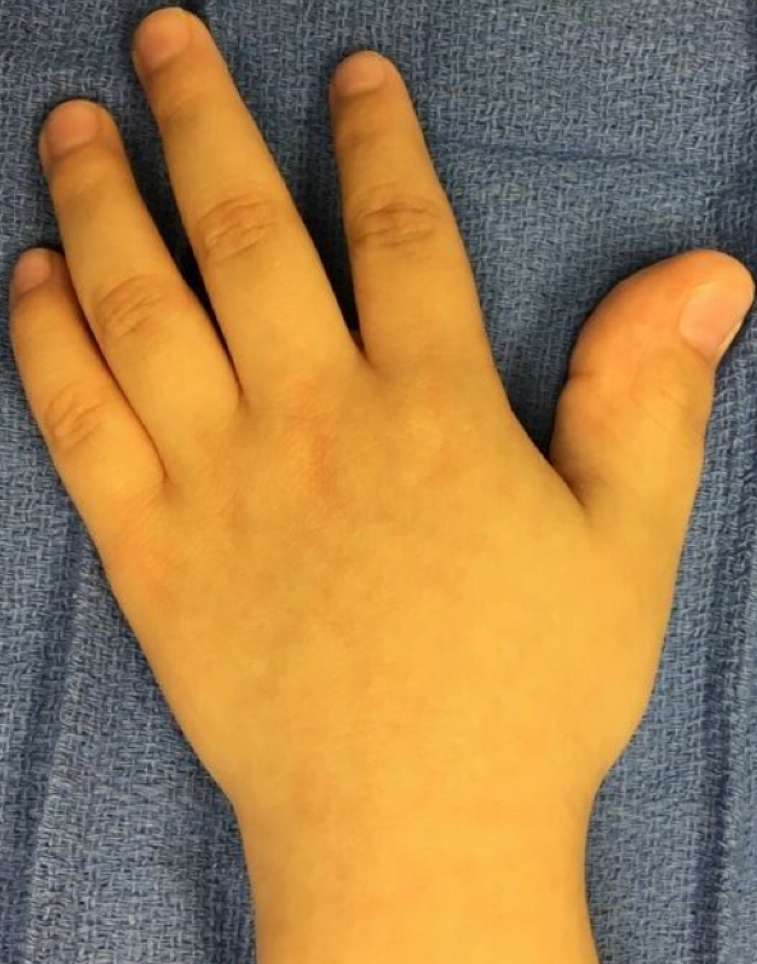

Supplement: Supplementary file 4 [file gox-7-e2483-s004.pdf]
